# Communication and re-use of chemical information in bioscience

**DOI:** 10.1186/1471-2105-6-180

**Published:** 2005-07-18

**Authors:** Peter Murray-Rust, John BO Mitchell, Henry S Rzepa

**Affiliations:** 1 Unilever Centre for Molecular Science Informatics, Department of Chemistry, University of Cambridge, Lensfield Road, Cambridge. CB2 1EW, UK; 2 Department of Chemistry, Imperial College London, SW7 2AY, UK

## Abstract

The current methods of publishing chemical information in bioscience articles are analysed. Using 3 papers as use-cases, it is shown that conventional methods using human procedures, including cut-and-paste are time-consuming and introduce errors. The meaning of chemical terms and the identity of compounds is often ambiguous. valuable experimental data such as spectra and computational results are almost always omitted. We describe an Open XML architecture at proof-of-concept which addresses these concerns. Compounds are identified through explicit connection tables or links to persistent Open resources such as PubChem. It is argued that if publishers adopt these tools and protocols, then the quality and quantity of chemical information available to bioscientists will increase and the authors, publishers and readers will find the process cost-effective.

## Introduction

In a previous article [1] we have argued the value of extracting the chemical information in bioscientific research, transforming it to XML and redisseminating it openly. The present article expands on the technical and cultural infrastructure required to support this. The technical aspects have been solved to proof-of-concept stage and we are starting to embark on experiments in the social domain. In this we thank BMC for inviting us to submit this and we present a model here which we believe could be attractive for bioscience publishers and their community.

We concentrate on the current publication of chemistry in bioscience. This includes:

1. mention of chemical compounds.

2. details of synthesis (in vivo and in vitro) of compounds.

3. proof of structure (spectra and analytical data).

4. Methods and reagents in bioscience bio-protocols

5. properties of compounds.

6. reactions and their properties, both in enzymes and enzyme-free systems.

This type of chemistry is very well understood and has a simple ontology which has not changed over decades[2]. Unlike much bioscience, where ontological tools are an essential part of reconciling the domain-dependent approaches, much chemistry has an implicitly agreed abstract description. The problems are primarily reconciling syntax and semantics. This is because chemists use abbreviated and lazy methods of communicating data, relying on trained readers to add information from the context. We have reviewed current problems of machine-understanding of chemistry[3] in a typical chemistry journal, many of which are perpetuated by the graphical orientation of conventional publishing houses. Here we take the view that a committed publishing house can create a cost-effective and human-tolerable system for authoring semantically correct chemistry in (bio)scientific documents.

We know from experience that Utopian visions do not sell themselves. The enormous and accepted value of the sequence and structures databases arose not from the demands of individual authors, but from wider communities of researchers, funders, and learned societies. Even now the deposition of protein structure data, without which journals will not generally accept a paper, is seen by some as a chore and at worst as the donation of information to competitors. Without that commitment and the resource, however, Structural Biology would not exist as a discipline. Here we present the following vision; that aggregated "small-molecule" chemical information, if deposited at publication, aggregated and disseminated, would be seen as worth paying the prices of inconvenience.

## Generic infrastructure

For this proposal we make some assumptions about the evolving informatics environment:

• The costs of archiving and maintaining scientific information can be now very much lower than some of the more traditional approaches. There will always be areas (patents, safety, reference data) where intensive human effort is required in the curation of data and where comprehensiveness is critical. This argument will be strongly made by the current chemical secondary publishers who show no signs of changing their business model. However bioscience has shown that informatics research is willing to balance quantity and quality and accepts that data is always used under *caveat emptor*.

• Much data is now completely captured instrumentally and can, in principle, be transmitted without syntactic loss. Crystallography has shown that experimental data (in the CIF format) can be directly submitted to the publisher. Moreover with the development of expert programs it is possible to review the data by machine and that this leads to higher quality than before. The global aggregation of current small-molecule crystal structures, without any secondary curators or publishers, can now meet almost all the needs of the community.

• Most current publicly funded chemical data is never published; loss varies between 80% (crystallography) and 99.9%. Much of this is due to the lack of simple technical and cultural protocols, which we address later.

• The primary cost is human time. Storage and CPU costs are trivial (for our domain). We show how the measures here, if adopted, would save all members of the community considerable time. They would also lead to the creation of greatly enhanced information resources.

• A variety of repositories will become available. In some communities (e.g. Physics and Computer Science) self-archiving of (p)reprints is universal but in others it is rare. Early adopters of Institutional repositories (IRs) are starting to mandate that the output of publicly funded scholarship is reposited, and we infer that, perhaps with sharing schemes, this will become quasi-mandatory. There is potential conflict with publishers' licenses, which we address below.

• There will be sufficient publishers in bioscience who are attracted by approaches like ours, and that this will create a critical mass. The benefits will be interoperable approaches to authoring (at present the technical requirements of some publishers are grotesque, i.e. hardcopy, strange formats, etc.).

• Openness. Our approach does not require Open Access, but does require that chemical data are Open.

• Willingness for bioscientists to take a lead in chemical informatics. Chemical information sources are manually aggregated and curated secondary publications whose philosophy has barely altered over 120 years. There is a large quasi-monopoly of a small number of large organisations who have no interest or inclination in changing their business models or adopting the vision of the Semantic Web. These new technologies, however, are ideally suited to our model and require only modest investment.

• Open or cheap tools for publishing structured documents (in XML) which can be customised for different domains. As XML becomes the universal technology for publication and interoperability, the community has access to them and will become trained in their use. As Open source components become more widespread it becomes possible to envisage scientific authoring tools which meet many requirements of the publication process.

We look to bioscience to take a lead in helping realise the following vision. On the positive we now believe that there are already enough Open tools and Open resources which with communal will among bioscience authors and publishers can make the vision attractive and cost-effective.

## Automatic capture of chemical information

Much chemical data is largely context-free in that it can be understood and recreated independently of the location or motivation. The primary data model is over 120 years old and was developed by Beilstein in the 19th century and comprises three components: the chemical compound, its properties and citations. A pure compound is described by an immutable structural formula and has precisely reproducible properties. There are qualifications (e.g. some properties may depend on the precise crystalline form) but it has served as the basis of a multimillion chemical information market, with the compound at the centre. Current thinking asserts that the biological action of a compound is, in principle, reproducible and predictable if the system is carefully enough replicated and the components understood. This is the central dogma of the chemically-based pharmaceutical industry.

Chemistry has a tradition of quality through properties and analysis, so every new compound (and many resynthesised ones) mentioned in the literature must be accompanied by measurements of properties to justify identity and purity. These facts are available, in text form, in the primary literature in which over a million new compounds are published annually. Because structure predicts properties, and because drug discovery is so difficult, the pharmaceutical industry tests many compounds for biological activity. It is therefore the primary financial engine for the chemical information industry.

### The components

Techniques for managing items 1–5 listed above such as aggregating chemical compounds, properties and for searching the results, are very well understood and can be easily made nearly automatic. Most of the information of benefit to the community exists on the authors' computers in machine-processable form. It can be automatically converted into fine-grained XML[4] with almost no loss. The chemist has electronic copies of molecular structures, spectra and properties whose semantics are extremely well understood and where a simple technical protocol for conversion to XML and hence publication can be created. To support this part of the data publication process we have created the XML-based Chemical Markup Language (CML)[5]. The primary information components (all of which are common and well understood) are:

#### • Molecular structure

A compound is described by a compositional formula (e.g. CH3OH for methanol) and a graphical structural formula ("2D diagram"). These descriptions are required in bioscience publications for new compounds or where scientific arguments are based on details of chemical structure. There are a few widely used standalone tools (mainly commercial) for drawing structures and calculating certain properties. They output a variety of machine-processable formats (MDL Molfiles, ChemDraw CDX files, and increasingly CML). The main challenge is that the output is designed for the sighted human reader and that semantics may be implicit, discussed below. The Open Source community is creating tools (e.g. JChempaint[6]) that will be valuable in authoring publications.

#### • Chemical entities and names

Many compounds have no explicit structures and are mentioned by names or identifiers. Where these relate to specific compounds (rather than generic such as "phenols") it is valuable to link them to a precise identification.

#### • Spectra

Many traditional formats (JCAMP and some manufacturers) are satisfactorily machine-processable, and we expect the XML-based AnIML[7] to be widely adopted by manufacturers.

#### • Crystal Structures

Relatively few small-molecule crystal structures are reported in bioscience publications, but when they are we have a workflow-driven system that extracts the data automatically and reposits it

#### • Molecular properties

These are required as proof-of-synthesis and use a small dictionary of properties[8]. Their publication is highly ritualised and we expect that a publisher-wide template for the submission of this information would be straightforward to compile and welcomed by many authors.

### Identifying compounds

The identification of chemical entities is the most valuable contribution that an author can make. In most cases s/he (as, say, the purchaser or creator of the materials) is the best judge of what was used. It is more considerably more difficult to identify compounds after publication as we show below.

We list possible methods of publishing the identity of compounds in machine-understandable form:

#### • Connection table

This is the most powerful method and we urge that every report of a chemical synthesis be accompanied by a connection table. It already exists in the authors' laboratory (in MOL, Chemdraw, SMILES, and increasingly as CML). It is rare that a pure molecule in the bioscience literature cannot be represented in this way. This is the single most important recommendation in this manuscript.

#### • Chemical structure diagram

This is a useful adjunct to a connection table (and some of the formats combine the two). Very occasionally (e.g. for catenanes, helicenes) a diagram is essential, but it should never be used instead of a connection table.

#### • InChI

For most compounds of bioscientific interest with known structure it is possible to generate a unique identifier using the new InChI (International Chemical Identifier)[9] from IUPAC. This has major advantages over non-semantic identifiers and Closed proprietary canonical identifiers such as SMILES[10]. In principle an InChI not only uniquely identifies the substance but also contains all the essential structural information. InChI in its current version (1.0) (and often other canonicalization schemes as well) has some significant limitations for systems with metal ions and ionic compounds, a situation which apparently will be addressed in a future InChI revision. Simple molecules such as e.g. cis-Platin are currently included in the handling, although the stereochemical aspects are *not *currently captured. Handling such aspects of e.g. metal-based drugs must clearly be a high priority in the development of InChI.

#### • Semantically free identifiers

These are provided by authorities (e.g. Chemical Abstracts, RTECS, PubChem, etc.). To be useful they should have an Open mechanism for their resolution (e.g. in PubChem), but this is often expressly forbidden. Thus Chemical Abstracts[11] forbids the public exposure of more than 0.1% of its content. Unless persistent Open machine-friendly resolution is available we deprecate the use of authority-controlled identifiers as unique IDs in primary publications. There are very few cases (e.g. zeolites) where identifiers are the best means of identification.

#### • Trivial ("Common") names

The structures of many compounds ("aspirin", "testosterone", "glycine"...) can only be found through lookup. In the past these names have been controlled in Closed collections but there are now an increasing number of Open lexicons of names:structures. The NCI led the way (220,000 names for ca 50,000 structures) and PubChem [12] has continued to develop this. If commercial suppliers make their catalogs Open then most of the common chemical names in scientific discourse can be automatically linked to connection tables.

#### • Systematic chemical names

Until now this has been a common means of transmitting chemical identity, but it now serves little purpose, although It may be required legally, e.g. for patents. Most chemists would prefer a structural diagram to a systematic name, and many regard name generation as a tedious chore. In principle IUPAC[2] chemical names obey a context-free grammar and there are complex rules for canonicalization. In practice most authors use a variety of shortcuts. This means that most compounds are reported with a variety of near synonyms (thus "2-hydroxy-toluene", "2-methyl-phenol" are semi-systematic variants for "1-hydroxy-2-methyl-benzene"). Free-text searching on chemical names has almost always low precision and often low recall. It is a common error to assume that deterministic grammars can parse any chemical name; in practice typographical errors, elisions and trivial fragments lower precision considerably. Some commercial tools are available but their algorithms are closed and little research has been done on their precision and recall. We suspect that they are composed of lexicons and heuristics but have no information on how they are maintained, especially in light of revisions of naming conventions.

### Issues with chemical names

Chemical names can be used with more or less specificity. Thus "1,4-dichlorobenzene" is unambiguous in any context. However there are several areas where more generic language is used. This can arise because:

• The name refers to a class of compounds, whose members have similar structures and/or properties: "steroids", "amino acids", "monosaccharides", "polychlorinated biphenyls".

• The substance is a mixture of compounds: "60–80 petrol", "xylenes", "the phospho-inositols"

• The substance has not been fully identified: "the estradiol monobenzoate was ..." (there are two possibilities)

• The stereochemistry is ambiguous. The possibilities (in decreasing order of merit) include:

*Stereochemistry is known and reported*.

*Stereochemistry is unknown and reported as such*.

*Stereochemistry is partially known and reported as such*.

Stereochemistry is not reported but is unknown

Stereochemistry is not reported but is known

Stereochemistry is partially reported but is completely known

Stereochemistry is reported and is wrong

"Glutamic acid" is an example of ambiguity through unspecified stereochemistry. Thus PubChem lists the three isomers (Table 1).

**Table 1:** Isomers of Glutamic Acid

	Name(s)
611	glutamic acid
33032	L-glutamic acid
23327	D-glutamic acid

A structure without any stereo information is more valuable than one with partial information of unknown quality. The InChI [9] is an extremely powerful tool here. We have recently shown that "staurosporine" reported in publications and suppliers catalogs contains many instances of incorrect stereochemistry, and some partially correct and incorrect. Given that this is a single substance, its structure and absolute configuration has been known for a considerable time there is no reason for using any structure other than PubChem CID: 44259

#### • Ionization

Protons are labile in aqueous systems and (for example) aminoacids can have several ionization states. The importance of ionization details varies;

"Acetic acid (0.1 M) was added..." [ionization state irrelevant]

Acetic acid forms a hydrogen-bonded dimer in the crystal. [single species, determinable by crystallography]

We computed the structure of glycine zwitterion acid (NH_3_^+^CH_2_CO_2_^-^) in the gas-phase [single species, distinct from NH_2_CH_2_CO_2_H]

"Glutamic acid is the most common excitatory neurotransmitter in the CNS" [irrelevant in macroscopic experiment, critical in modelling action at receptor]

#### • Tautomerism

**Figure 1:**
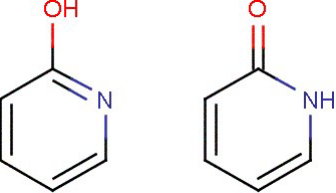
Tautomers of Hydroxypyridine

Many neutral compounds, particularly with heteroatoms have mobile hydrogens in solution. Thus 2-hy-droxy pyridine (see Figure 1) exists as both forms with very rapid interchange. PubChem (as with many other systems) lists them as the same compound (CID8871) and gives the many synonyms including "2(1H)-Pyridinone" and "2-HYDROXY-PYRIDINE". InChI[9] has an extensive system for detecting tautomerism in compounds with heteroatoms, but does not yet address carbon compounds (e.g. CH2=CHOH as a tautomer of ethanal (acetaldehyde, CH3-CH=O).

#### • Imprecise or polysystemic use

This often occurs when a chemical entity is incorporated into a larger system

"This polysaccharide has a high mannose content" means "... contains many mannosyl fragments ..."

"HIV protease has a catalytic aspartic acid..." means "... an aspartyl residue ..."

The preceeding discussion shows how ambiguity and loss of information can occur if structured procedures are not followed. The examples in Table 2 show some suggested approaches to markup which can re-capture much of the information loss described above. The last example references a generic name, monosaccharide, in the IUPAC guide[2] to organic nomenclature with a suggested use of identifiers.

**Table 2:** Examples of approaches to chemical Identification.

**Prose description**	**More precise suggested naming using the CML[5] approach**	Type of information
*Acetaldehyde *has a general narcotic action	<p><cml:molecule> <cml:identifier convention="iupac:inchi">1/C2H4O/c1-2-3/h2H,1H3</cml:identifier> <cml:identifier convention="pubchem:CID">177</cml:identifier> </cml:molecule> has a ...</p>	precise, redundant
*Benzo(a)pyrene *is a potent mutagen and carcinogen	<p><cml:molecule><cml:identifier convention="pubchem:CID">2336</cm l:identifier></cml:molecule> is a ...</p>	precise
*glycine *(1 mmol) was added ...	<p><cml:molecule title="glycine"><cml:identifier convention="iupac:inchi">1/C2H5NO 2/c3-1-2(4)5/h1,3H2,(H,4,5)</cml: identifier></cml:molecule> is a ...</p>	hydrogens mobile
calculations on *glycine zwitterion...*	<p><cml:molecule title="g><cml:identifier convention="pubchem:CID">InChI=1/C2H5NO2/c3-1-2(4)5/h1H3,3H2</cml: identifier></cml:molecule> is a ...</p>	hydrogens precise
... a monosaccharide transporter...	<p>a <cml:molecule title="monosaccharide"><cml:ident ifier convention="iupac:carbohydrate">2 -Carb-2</cml:identifier></cml:mol ecule> transporter ...</p>	Data

## Case studies

In this second section, we take 3 articles from BMC publications and show the success and problems of extracting chemistry in machine-understandable form. These have been randomly selected and do not necessarily reflect the average quality of BMC publications. We note that in our other studies of chemical text very few publications were error-free.

### Case study 1: Identification of compounds in discourse and reagents in methods[13]

The abstract is typical of the discourse:

#### Background

*Recent studies indicate that the G protein-coupled receptor (GPCR) signaling machinery can serve as a direct target of reactive oxygen species, including nitric oxide (NO) and S-nitrosothiols (RSNOs). To gain a broader view into the way that receptor-dependent G protein activation – an early step in signal transduction – might be affected by RSNOs, we have studied several receptors coupling to the Gi family of G proteins in their native cellular environment using the powerful functional approach of [35S]GTPgammaS autoradiography with brain cryostat sections in combination with classical G protein activation assays*.

#### Results

*We demonstrate that RSNOs, like S-nitrosoglutathione (GSNO) and S-nitrosocysteine (CysNO), can modulate GPCR signaling via reversible, thiol-sensitive mechanisms probably involving S-nitrosylation. RSNOs are capable of very targeted regulation, as they potentiate the signaling of some receptors (exemplified by the M2/M4 muscarinic cholinergic receptors), inhibit others (P2Y12 purinergic, LPA1lysophosphatidic acid, and cannabinoid CB1 receptors), but may only marginally affect signaling of others, such as adenosine A1, μ-opioid, and opiate related receptors. Amplification of M2/M4 muscarinic responses is explained by an accelerated rate of guanine nucleotide exchange, as well as an increased number of high-affinity [35S]GTP?S binding sites available for the agonist-activated receptor. GSNO amplified human M4 receptor signaling also under heterologous expression in CHO cells, but the effect diminished with increasing constitutive receptor activity. RSNOs markedly inhibited P2Y12 receptor signaling in native tissues (rat brain and human platelets), but failed to affect human P2Y12 receptor signaling under heterologous expression in CHO cells, indicating that the native cellular signaling partners, rather than the P2Y12 receptor protein, act as a molecular target for this action*.

#### Conclusion

*These in vitro studies show for the first time in a broader general context that RSNOs are capable of modulating GPCR signaling in a reversible and highly receptor-specific manner. Given that the enzymatic machinery responsible for endogenous NO production is located in close proximity with the GPCR signaling complex, especially with that for several receptors whose signaling is shown here to be modulated by exogenous RSNOs, our data suggest that GPCR signaling in vivo is likely to be subject to substantial, and highly receptor-specific modulation by NO-derived RSNOs*.

**Figure 2:**
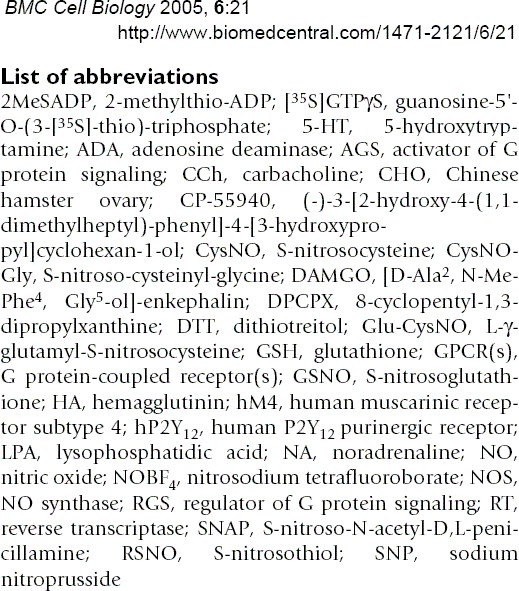
Abbreviations used in reference 13.

The above contains reference to a considerable numbers of individual compounds. The authors helpfully publish a table of abbreviations to assist in the compound identification process (Figure 2). Using this as our data, we have attempted to identify (Table 3) the "small-molecules" mentioned in the discourse. Using PubChem and occasional suppliers catalogs, the elapsed real time was about 1 hour. It can be seen that of 19 molecules, 15 were identified without problems or error, 2 were not (CysNOGly and Glu-CysNO) and 2 required additional expertise by the reader. We estimate that it would take an author the same amount of time to add PubChem IDs for novel compounds and much less time if they were in common use in their laboratory.

**Table 3:** Identification of Small-molecules noted in Ref. 14

**abbreviation**	**author name**	**PubChem ID**	**Notes**
			Not found directly in PubChem. Located in supplier's catalog. Synonym from that found in PubChem
2MeSADP	2-methylthio-ADP	[121990]	
5-HT	5-hydroxytryptamine	5202	
CCh	carbacholine	521353	
CP-55940	(-)-3-[2-hydroxy-4-(1,1-dimeth ylheptyl)-phenyl]-4-[3-hydroxyp ropyl]cyclohexan-1-ol	104895	IUPAC: 5-(1,1-dimethylheptyl)-2-[5-hydroxy-2-(3-hydroxyprop yl)cyclohexyl]-phenol
CysNO	S-nitrosocysteine	39933	
CysNOGly	S-nitroso-cysteinyl-glycine		Text search on PubChem found wrong compound. Not found in major supplier
DAMGO	[D-Ala2, N-Me-Phe4, Gly5-ol]-enkephalin	104742	
DPCPX	8-cyclopentyl-1,3-dipropylxanthi ne	1320	
Glu-CysNO	L-?-glutamyl-S-nitrosocysteine		Identity unresolved
GSH	glutathione	745	
GSNO	S-nitrosoglutathione	104858	
LPA	lysophosphatidic acid	3987	
NA	noradrenaline	951	PubChem CID covers both racemic and d-enantiomer
NO	nitric oxide	84878	PubChem also lists 945 (with incorrect formula HNO) as nitric oxide
NOBF4	nitrosodium tetrafluoroborate	151929	Paper has a typographical error for "nitrosonium". Structure in PubChem is wrong (formula should be NO^+^BF_4_^-^, not H_2_NO^+^.BF_4_^-^)
SNAP	S-nitroso-N-acetyl-D,L-penicill amine	5231	PubChem does not list stereochemistry
RSNO	S-nitrosothiol		Appears to be a generic compound (R-S-N=O)
SNP	sodium nitroprusside	26256	

It is only a little additional effort to convert each molecule to a more formal description expressed in e.g. CML[5] and which can carry not only an atom connection table and the corresponding InChI identifer, but also molecule "meta-data" describing the provenance of the information:

<cml:molecule xmlns:cml="" title="carbacholine">

<cml:metadataList title="generated automatically from Openbabel">

<cml:metadata name="dc:creator" content="OpenBabel version 1-100.1"/>

<cml:metadata name="dc:description" content="Conversion of legacy filetype to CML"/>

<cml:metadata name="dc:identifier" content="InChI"/>

<cml:metadata name="dc:content"/>

<cml:metadata name="dc:rights" content="open"/>

<cml:metadata name="dc:type" content="chemistry"/>

<cml:metadata name="dc:contributor" content="rzepa"/>

<cml:metadata name="dc:creator" content="Openbabel V1-100.1"/>

<cml:metadata name="dc:date" content="Tue May 17 12:02:50 BST 2005"/>

<cml:metadata name="cmlm:structure" content="yes"/>

</cml:metadataList>

<cml:identifier convention="iupac:inchi">InChI=1/C6H14N2O2.ClH/c1-8(2,3)4-5-10-6(7)9;/h4-5H2,1-3H3,(H-,7,9);1H</cml:identifier>

<cml:atomArray atomID="a1 a2 a3 a4 a5 a6 a7 a8 a9 a10 a11 a12 a13"

    elementType="N C C O C O N C C C H H Cl"

    formalCharge="1 0 0 0 0 0 0 0 0 0 0 0 -1"

    x2="-1.892900 -1.178500 -0.464000 0.250500 0.964900 0.964900 1.761800

-2.305400 -2.476300 -1.480400 2.174300 2.476300 -1.921800"

    y2="0.415300 0.827800 0.415300 0.827800 0.415300 -0.409700 0.628800 1.129800

-0.168000 -0.299200 1.343300 0.216300 -1.343300"/>

<cml:bondArray atomRef1="a1 a1 a1 a1 a2 a3 a4 a5 a5 a7 a7"

       atomRef2="a2 a8 a9 a10 a3 a4 a5 a6 a7 a11 a12"

       order="1 1 1 1 1 1 1 2 1 1 1"/>

</cml:molecule>

Such molecular datuments can be embedded in any XML-based document in a manner which can if needed survive e.g. journal production processes, and where the molecular information can be extracted and re-used at any stage.

### Case study 2: Identity and properties of synthesised compounds[14]

Our critique of the chemistry requires context, given by the abstract:

#### Abstract background

*Kynureninase is a key enzyme on the kynurenine pathway of tryptophan metabolism. One of the end products of the pathway is the neurotoxin quinolinic acid which appears to be responsible for neuronal cell death in a number of important neurological diseases. This makes kynureninase a possible therapeutic target for diseases such as Huntington's, Alzheimer's and AIDS related dementia, and the development of potent inhibitors an important research aim*.

#### Results

Two new kynurenine analogues, 3-hydroxydesaminokynurenine and 3- methoxydesami-nokynurenine, were synthesised as inhibitors of kynureninase and tested on the tryptophan-induced bacterial enzyme from Pseudomonas fluorescens, the recombinant human enzyme and the rat hepatic enzyme. They were found to be mixed inhibitors of all three enzymes displaying both competitive and non competitive inhibition. The 3-hydroxy derivative gave low Ki values of 5, 40 and 100 nM respectively. [...]

#### Conclusion

For kynureninase from all three species the 2-amino group was found to be crucial for activity whilst the 3-hydroxyl group played a fundamental role in binding at the active site presumably via hydrogen bonding. The potency of the various inhibitors was found to be species specific. The 3-hydroxylated inhibitor had a greater affinity for the human enzyme, consistent with its specificity for 3-hydroxykynurenine as substrate, whilst the methoxylated version yielded no significant difference between bacterial and human kynureninase. [...]

We note that "quinolinic acid" has four mentions in the text, but its formula is not given. We took roughly three minutes to identify CID1066 in PubChem, with the additional useful information (from Medline/MeSH):

A metabolite of tryptophan with a possible role in neurodegenerative disorders. Elevated CSF levels of quinolinic acid are correlated with the severity of neuropsychological deficits in patients who have AIDS

The name "3-hydroxydesaminokynurenine" [the synthesized compound (4) presents a more serious problem. Although the structure is given in a diagram, the stereogenic centre is not marked. It would be a reasonable assumption that "kynurenine" refers to a natural product which is only found in one enantiomeric form and "desamino" was also chiral. Careful reading (requiring chemical expertise) showed that the authors had probably synthesised a racemic mixture, since they started with achiral compounds and did not report chiral reagents or a resolution step. The compound should have been reported as (R/S)-3-hydroxydesaminokynurenine or (much better) as the IUPAC-like name "IUPAC Name: (R/S) 2-amino-4-(3-hy-droxy-phenyl)-4-oxo-butanoic acid". Indeed many referees and editors would have insisted on this specification. In the event, as we show below, this is not the reported compound!

The tools we are proposing would immediately have queried both these concerns at time of authoring and, had they been available to the technical editor would have produced a more useful and more easily readable paper.

**Figure 3:**
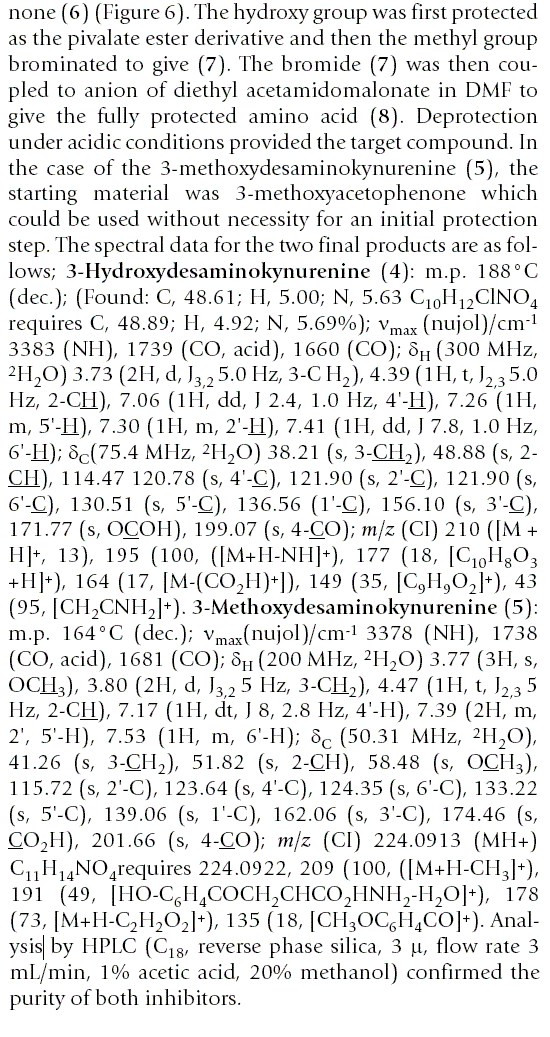
A linear text-based description of experimental detail and data taken from Ref. 14.

The publication of the synthesis or re-synthesis of compounds must be accompanied by analytical and property data to prove purity and identitity. The ritualistic presentation shown below (Figure 3) as copied from the manuscript is entirely typical of most chemical publications. Note that it is visually challenging to read and this is entirely due to the publisher's requirements of using a system designed to save paper rather than communicate useful information.

For each compound this compressed information is (manually) created from some or all of:

1. An elemental analysis (probably in machine-understandable form)

2. A calculated composition for the compound (machine understandable)

3. An infrared spectrum (machine understandable)

4. A ^1^H NMR spectrum (machine understandable)

5. A ^13^C NMR spectrum (machine understandable)

6. A low resolution mass spectrum (machine understandable)

7. A high resolution mass spectrum (machine understandable)

**Figure 4:**
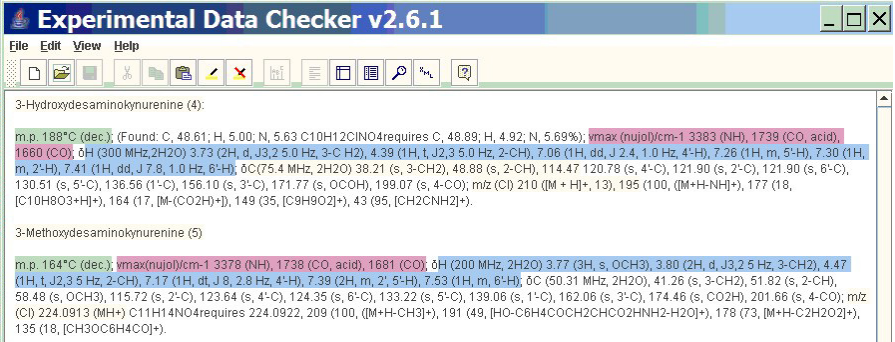
OSCAR output from the text-based description in Ref 14.

For the publication, the authors have to measure peak heights from the spectrum (possibly with a ruler), and transcribe them to a Word or PDF format, probably by typing the values or cut-n-pasting them. We have developed an Open Source robot (OSCAR)[8] which can understand this data if it is syntactically correct, and the result is shown in Figure 4:

The coloured parts are those that adhere to the publication guidelines. We found 7 changes that had to be made to the punctuation (missing punctuation, syntactic variation is common in many chemical papers). OSCAR can then understand and check the data. For compound [4] OSCAR produces the errror message:

There are fewer H atoms by NMR integration (7) than there are by elemental analysis (12)

This is acceptable because there are exchangeable groups. However OSCAR also gives the error flag:

There are more C-NMR environments (11) than there are C atoms from elemental analysis (10).

as it found the string "114.47 120.78". We also do not understand this and it may be an error (or it could be a solvent peak or other impurity). OSCAR also had problems interpreting the chemical formula: "C_11_H_14_NO_4_" which in fact turns out to be a charged species. In fact the compounds are poorly identified. They appear to be not the aminoacids "3-Hydroxydesami-nokynurenine (4)" and "3-Methoxydesaminokynurenine (5)" but their hydrochloride salts. This is not a trivial error; the melting points and infrared spectra of the parents and their salts will be significantly different and would cause errors if transcribed unthinkingly from the paper.

**Figure 5:**
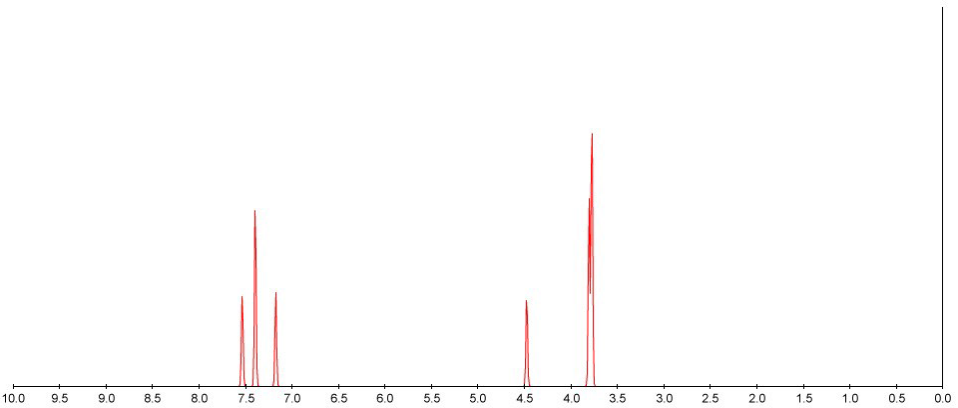
OSCAR generated spectrum of analytical information reported in Ref 14.

Even with OSCAR it took one of us ca 45 minutes to make sure that the above analysis was correct. From several anecdotal conversations with typical authors we estimate that it took about 2 hours to prepare this part of the submission; a thorough reviewer might take 0.5 hour to decipher it. All of this is unnecessary if the original connection tables, spectra and analytical data were made available in uncorrupted form. As it is, much of the original data is lost; using the reported peaks OSCAR does its best to recreate what the spectrum might have looked like (Figure 5). Precise peak shapes and traces of impurities are lost in this representation.

### Case study 3. Identity of compounds and preservation of calculations[15]

Here too a number of small-molecules are reported without formulae;

*Background [...] Phenols and anilines are generally recognized as substrates of the heme peroxidases (donor: H2O2 oxidoreductases EC 1.11.17). The peroxidases catalyze oxidation of the substrates by hydrogen peroxide or alkyl peroxides, usually but not always, via free-radical intermediates *[1,2]. *Nonphenolic compounds, such as indole-3-acetic acid, phenylenediamines, ferrocenes, phenothiaz-ines, phenoxazines, have also been investigated as peroxidase substrates *[2-5]. *Steady-state kinetics of peroxidase action has been described as a ping-pong scheme with compound I and compound II formation *[1].

**Figure 6:**
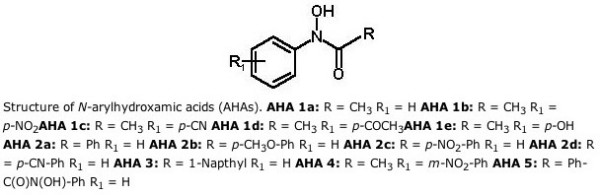
Structure diagram reported in Ref 15.

This paper also has issues with the identity of compounds (Figure 6). This is again a visually unacceptable format dictated by the prevailing business model of chemical publishing. Note "Napthyl" is misspelt, presumably because it has been (mis)typed by the authors, which would give unnecessary problems to chemical text-mining robots. Worse, the identity of AHA5 is genuinely unclear, in that the connection could be to either of the phenyl groups in the fragment: "Ph-C(O)N(-OH)-Ph". BHA (also described elsewhere by "benzhydroxamic acid") has no structural or compositional formula. Worse, BHA in the PDB ligand collection refers to 2-hydroxy-4-amino-benzoic acid (a completely different compound); "benzhydroxamic acid" has code BHO.

Another section of this article describes various computational modelling techniques applied to these molecules; here we can assume that the authors had precise coordinates for all the computed species available at the end of the research, although none of this data is actually made available via the final published article. Some of this data is used to drive a docking program, which itself implies a protocol used to specify various run-time parameters. Some of these are declared in the article, many probably default to values set internally within the program. There are also ambiguities in the declared computational protocol:

*The optimized geometry of molecules was used for energies and charges calculations with a 6-31G basis set using RHF and B3PW91 (Density Functional Theory)*.

Here, the RHF and the B3PW91 protocols are mutually exclusive; either one or the other could have been used, but not in combination. Mapping either protocol to e.g. the appropriate input for the program package used can also be a challenge for anyone not totally familiar with the program; program manuals are still designed largely for human rather than machine use. Such ambiguities, and lack of data, make repetition of the modelling more difficult for others.

## A proposed infrastructure

It should now be clear that the current system of communicating chemistry (which is common to all publishers and all disciplines) is inefficient, costly, lossy, and of questionable quality. We present a new XML-based approach which we show:

• takes less time

• conveys more information

• is easier to read

• allows published data to be aggregated and re-used

We note that when starting to draft a publication the author already has

**• free text (A) **(probably in handwritten form)

**• properties (B) **(probably handwritten form)

**• spectra (C) **(probably in digital form)

**• molecules (D) **(probably in MOL or ChemDraw files)

**Figure 7:**
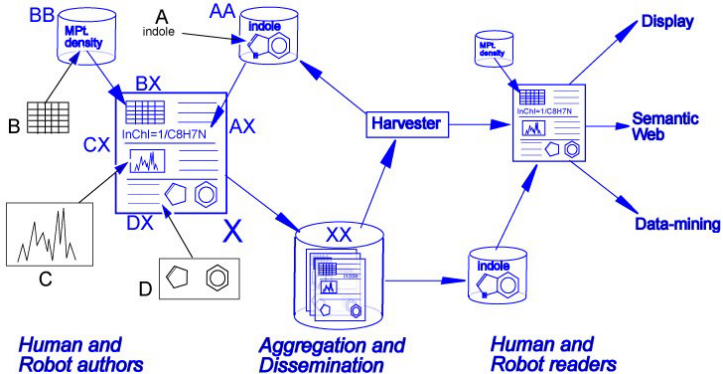
Data-flow illustrating the use of XML.

Electronic lab notebook technology is not well advanced in chemistry; our architecture would provide a good method for preserving conventional data. It looks as shown in Figure 7 (blue = XML):

The author would then use a tool which can manage structured XML documents and provide normal textual support (spellchecks, etc.). There are 4 additional tools required to support chemical information:

**• A**. Chemical lexical tool(**AA**) which can (a) parse free text(**A**) for possible compound names (b) look them up or (c) parse them to create connection table and (d) insert a reference (**AX**) to the lexicon in the text, e.g.:

... When foobarone is broken down, the presence of indole can be detected ...

might be marked up as

... When <cml:molecule name="foobarone" dictRef="natprod:foobarone"/> is broken down, the presence of <cml:molecule>

<identifier convention="iupac:inchi" title="indole">1/C8H7N/c1-2-4-8-7(3-1)5-6-9-8/h1-6H,9H</identifier>

</cml:molecule>

indole can be detected ...

**• B**. A controlled vocabulary (**BB**) of property types is used in a template to capture properties (**B**) and create a CML table (**BX**), e.g.

yield(93%), M.Pt. 273-275°C

becomes

<cml:list>

    <cml:property dictRef="cml:yield">

       <cml:scalar units="cml:percent">93%lt;/cml:scalar>

    </cml:property>

    <cml:property dictRef="cml:mpt">

       <cml:scalar units="cml:celsius" minValue="273" maxvalue="275"/>

    </cml:property>

</cml:list>

**• C**. Spectra in legacy format (**C**) are automatically converted to CMLSpect or AniML (**CX**).

**• D**. Molecules created in a conventional editor are either emitted in CML (DX) or automatically converted from legacy (**D**) .

The result is a single structured XML "datument"[16] containing fine-grained markup of facts (molecules, measurements, properties, chemical names). This datument can be used to create derivatives such as the "full-text" or the "supplemental data". The complete datument (if Open) or the "data" if not is then reposited (**XX**) where it can be harvested. New compounds with their names are fed back into the lexicon and all compound/property data is available for datamining and computational re-use (e.g. for further in silico prediction. A human or robot reader has access to the same lexicons and dictionaries as the author so that the semantics and ontology of authoring are the same as those of reading (and of preservation).

### Metadata and rights

The social aspects of metadata and rights were addressed in (1). To meet these we place special emphasis on the XML and its metadata. Fine-grained XML (e.g. <scalar>...</scalar> or <molecule>...</molecule> represents facts which can be identified as Open and not the property of the publisher. Hyperlinks and structure for semantics (e.g. identification of compounds in PubChem) are also Open. Tools such as XSLT can then extract the factual, non-copyrightable information with little technical problem. Rights should be explicitly marked up. If the publisher supports Open Access [17] and also Open Data then it is valuable to label the appropriate components with Open licenses, such as the RDF metadata provided by Creative Commons. It is also possible to preserve authors' moral rights and provenance of data re-used within the paper (e.g. spectra of molecules or coordinates of protein structures).

## Realising the vision

The transition to this architecture will have a cost, so short term-benefits are particularly attractive. Moreover most of the parties are not used to a communal approach (pressures are normally per-institution and per-publisher).

### Costs

• Time lost in understanding and changing to a new system.

• New tools might cost money, or have to come from non-centralised budgets

• Only supported by a subset of publishers

• Communal deposition of data goes against the secretive culture

• Publishers have to invest in new system and react to community expectations

### Benefits

• Open Access and Open Data[16]

• Greater quality in publications

• Data in theses and papers can be interchanged

• Greater readability, usability and innovation in publications

• Repository provides complete data record for laboratory, institution and world

• Modern informatics tools allow new types of search and aggregation

• Considerable time-savings during publication

• More efficient publishing reduces author frustration and time to publication

• and most importantly the arrival of the Scientific Semantic Web, allowing robots to read and take action on publications.

The benefits should also be clear for most individuals and organisations:

**• funders **can ensure a much higher of dissemination of funded data will be available

**• institutions **mandate a greater proportion of funded science published; better visibility and preservation

**• researchers **spend less time on unproductive operations

**• reviewers **have easier access to background ontology of data in documents

**• editors **get greater automation

**• publishers **are relieved of need to archive supplemental data

**• readers **have information prosthetics for easier and faster reading

**• librarians **develop one of the best early repository applications in the digital age

### Potential

**Figure 8:**
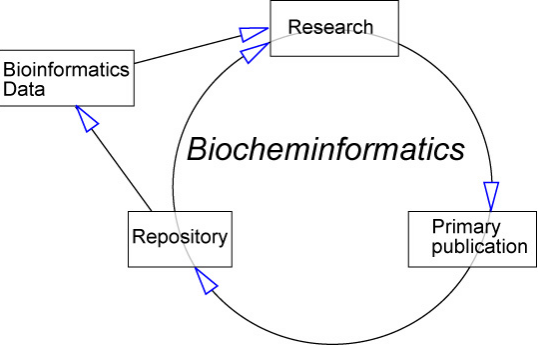
A Biocheminformatics Cycle.

Because the chemical information is structured we now have a biocheminformatics cycle (this term – with spelling as here -is in modest use. We suggest its adoption to describe the management of chemical information in biosciences and not just in biochemistry) where, for the first time, large scale robotic data analysis can take place (Figure 8).

The data in the research (laboratory, *in silico*, or both) are published in a lossless manner. Molecules and their properties have unique identifiers as described above and can be integrated into mainstream bioinformatics in the same manner as collections such as PubChem, MSDChem (at EBI), KEGG, etc. They will bring the added value of consistently captured property data and spectra. We also expect that many in silico properties will then be systematically added.

### Compliance and adoption

The current dissemination of data through publishers is largely unsatisfactory. Some publishers, such as the International Union of Crystallography, see it as core business and others in the biosciences agree to link to international databanks. Many publishers allow the deposition of factual "supplementary data" but our experience with mainstream chemical publishers is that it is an unwelcome chore, poorly resourced and maintained, and with virtually no quality control or curation over the content. We believe that many publishers would welcome a model where they were no longer involved in data repositing.

The introduction of structured authoring tools will help this process considerably. Templates can be created for the chemical components described above and where the information exists in XML (connection tables, spectra, properties) it should be as easy as for committed authors as using a semantically void tool (e.g. Word). Where information needs to be converted from legacy formats we have created Open Web Services which publishers (and authors) may clone and customise. The main technical challenge will be the management of chemical names in free text.

## Conclusions and the future

The analysis presented here introduces the basic concepts of chemistry in bioinformatics. Many areas remain to be addressed; we briefly describe two below which have immediate application.

### Reactions

Chemical reactions are very patchily abstracted from the literature and the products are almost always closed. The motivation for the primary publication of reactions in bioscience includes:

1. record of synthesis of compound and proof thereof

2. record of an experimental protocol (e.g. biotinylation)

3. record of a biochemical reaction, including xenobiotic processes

4. description of systems biochemistry (coupled reaction pathways)

5. understanding of an enzyme mechanism

CMLReact (an extension of CML) has been created[18] to support these catagories of reaction. Items 1-2 require identical support as in mainstream chemistry (e.g. in journals supporting organic synthesis). Item 3 can be supported by CMLReact though there is little current experience. Item 4 is supported by SBML[19] and efforts such as BioPAX[20] (in which CML is a tool). Item 5 is particularly exciting and exemplified by our MACiE database where 150+ enzymes with 3D structures and proposed mechanisms have been collected[21]. Currently the abstraction is manual and expensive, but if the ideas in the current paper are implemented we shall present an extension whereby mechanisms can be relatively cheaply captured at source. This would be a major new resource in bioinformatics.

### Evaluation metrics

The primary motivation for a publication, of course, is citability and the technology we describe raises the fear among chemists that the data in it might actually be read, analysed and re-used. However it also raises the vision of changing the "citation economy" (which values market perception) to a "reuse economy" where the data in an article (or as we prefer, a "datument") are valued by how often they are re-used.

## Supplementary Material

Additional File 1 Click here for file
