# Supplementary material for: Communication and re-use of chemical information in bioscience
Source: BMC Bioinformatics. 2005 Jul 18;6:180. doi: 10.1186/1471-2105-6-180 (PMC1187874; doi:10.1186/1471-2105-6-180)
Supplement: Additional File 1 [file 1471-2105-6-180-S1.html]

Communication and re-use of chemical information in
bioscience


# Communication and re-use of chemical information in bioscience

## Peter Murray-Rust1, John B.O. Mitchell1 and Henry S. Rzepa2

1 Unilever Centre for Molecular Science
Informatics, Department of Chemistry, University of Cambridge,
Lensfield Road, Cambridge. CB2 1EW, UK.  
2Department of Chemistry, Imperial College London,
SW7 2AY, UK.

## Abstract

The current methods of publishing chemical information in
bioscience articles are analysed. Using 3 papers as use-cases,
it is shown that conventional methods using human procedures,
including cut-and-paste are time-consuming and introduce
errors. The meaning of chemical terms and the identity of
compounds is often ambiguous. valuable experimental data such
as spectra and computational results are almost always omitted.
We describe an Open XML architecture at proof-of-concept which
addresses these concerns. Compounds are identified through
explicit connection tables or links to persistent Open
resources such as PubChem. It is argued that if publishers
adopt these tools and protocols, then the quality and quantity
of chemical information available to bioscientists will
increase and the authors, publishers and readers will find the
process cost-effective.

## Introduction

In an accompanying paper1 we have argued the
value of extracting the chemical information in bioscientific
research, transforming it to XML and redisseminating it openly.
The present article expands on the technical and cultural
infrastructure required to support this. The technical aspects
have been solved to proof-of-concept stage and we are starting
to embark on experiments in the social domain. In this we thank
BMC for inviting us to submit this and we present a model here
which we believe could be attractive for bioscience publishers
and their community.

We concentrate on the current publication of chemistry in
bioscience. This includes:

1. mention of chemical compounds.
2. details of synthesis (in vivo and in vitro) of
   compounds.
3. proof of structure (spectra and analytical data).
4. Methods and reagents in bioscience bio-protocols
5. properties of compounds.
6. reactions and their properties, both in enzymes and
   enzyme-free systems.

This type of chemistry is very well understood and has a
simple ontology which has not changed over decades2.
Unlike much bioscience, where ontological tools are an
essential part of reconciling the domain-dependent approaches,
much chemistry has an implicitly agreed abstract description.
The problems are primarily reconciling syntax and semantics.
This is because chemists use abbreviated and lazy methods of
communicating data, relying on trained readers to add
information from the context. We have reviewed current problems
of machine-understanding of chemistry3 in a typical
chemistry journal, many of which are perpetuated by the
graphical orientation of conventional publishing houses. Here
we take the view that a committed publishing house can create a
cost-effective and human-tolerable system for authoring
semantically correct chemistry in (bio)scientific
documents.

We know from experience that Utopian visions do not sell
themselves. The enormous and accepted value of the sequence and
structures databases arose not from the demands of individual
authors, but from wider communities of researchers, funders,
and learned societies. Even now the deposition of protein
structure data, without which journals will not generally
accept a paper, is seen as at best a chore and at worst as the
donation of information to competitors. Without that commitment
and the resource, however, Structural Biology would not exist
as a discipline. Here we present the following vision; that
aggregated "small-molecule" chemical information, if deposited
at publication, aggregated and disseminated, would be seen as
worth paying the prices of inconvenience.

## Generic Infrastructure

For this proposal we make some assumptions about the
evolving informatics environment:

- The costs of archiving and maintaining scientific
  information can be now very much lower than some of the more
  traditional approaches. There will always be areas (patents,
  safety, reference data) where intensive human effort is
  required in the curation of data and where comprehensive is
  critical. This argument will be strongly made by the current
  chemical secondary publishers who show no signs of changing
  their business model. However bioscience has shown that
  informatics research is willing to balance quantity and
  quality and accepts that data is always used under *caveat
  emptor*.
- Much data is now completely captured instrumentally and
  can, in principle, be transmitted without syntactic loss.
  Crystallography has shown that experimental data (in the CIF
  format) can be directly submitted to the publisher. Moreover
  with the development of expert programs it is possible to
  review the data by machine and that this leads to higher
  quality than before. The global aggregation of current
  small-molecule crystal structures, without any secondary
  curators or publishers, can now meet almost all the needs of
  the community.
- Most current publicly funded chemical data is never
  published; loss varies between 80% (crystallography) and
  99.9%. Much of this is due to the lack of simple technical
  and cultural protocols, which we address later.
- The primary cost is human time. Storage and CPU costs are
  trivial (for our domain). We show how the measures here, if
  adopted, would save all members of the community considerable
  time. They would also lead to the creation of greatly
  enhanced information resources.
- A variety of repositories will become available. In some
  communities (e.g. Physics and Computer Science)
  self-archiving of (p)reprints is universal but in others it
  is rare. Early adopters of Institutional repositories (IRs)
  are starting to mandate that the output of publicly funded
  scholarship is reposited, and we infer that, perhaps with
  sharing schemes, this will become quasi-mandatory. There is
  potential conflict with publishers' licenses, which we
  address below.
- There will be sufficient publishers in bioscience who are
  attracted by approaches like ours, and that this will create
  a critical mass. The benefits will be interoperable
  approaches to authoring (at present the technical
  requirements of some publishers are grotesque (hardcopy,
  strange formats, etc.)).
- Openness. Our approach does not require Open Access, but
  does require that chemical data are Open.
- Willingness for bioscientists to take a lead in chemical
  informatics. Chemical information sources are manually
  aggregated and curated secondary publications whose
  philosophy has barely altered over 120 years. There is a
  large quasi-monopoly of a small number of large organisations
  who have no interest or inclination in changing their
  business models or adopting the vision of the Semantic Web.
  These new technologies, however, are ideally suited to our
  model and require only modest investment.
- Open or cheap tools for publishing structured documents
  (in XML) which can be customised for different domains. As
  XML becomes the universal technology for publication and
  interoperability, the community has access to them and will
  become trained in their use. As Open source components become
  more widespread it becomes possible to envisage scientific
  authoring tools which meet many requirements of the
  publication process.

We look to bioscience to take a lead in helping realise the
following vision. On the positive we now believe that there are
already enough Open tools and Open resources which with
communal will among bioscience authors and publishers can make
the vision attractive and cost-effective.

## Automatic capture of chemical information

Much chemical data is largely context-free in that it can be
understood and recreated independently of the location or
motivation. The primary data model is over 120 years old and
was developed by Beilstein in the 19th century and comprises
three components: the chemical compound, its properties and
citations. A pure compound is described by an immutable
structural formula and has precisely reproducible properties.
There are qualifications (e.g. some properties may depend on
the precise crystalline form) but it has served as the basis of
a multimillion chemical information market, with the compound
at the centre. Current thinking asserts that the biological
action of a compound is, in principle, reproducible and
predictable if the system is carefully enough replicated and
the components understood. This is the central dogma of the
chemically-based pharmaceutical industry.

Chemistry has a tradition of quality through properties and
analysis, so every new compound (and many resynthesised ones)
mentioned in the literature must be accompanied by measurements
of properties to justify identity and purity. These facts are
available, in text form, in the primary literature in which
over a million new compounds are published annually. Because
structure predicts properties, and because drug discovery is so
difficult, the pharmaceutical industry tests many compounds for
biological activity. It is therefore the primary financial
engine for the chemical information industry.

### The components

Techniques for managing items 1-5 listed above such as
aggregating chemical compounds, properties and for searching
the results, are very well understood and can be easily made
nearly automatic. Most of the information of benefit to the
community exists on the authors' computers in
machine-processable form. It can be automatically converted
into fine-grained XML4 with almost no loss. The
chemist has electronic copies of molecular structures, spectra
and properties whose semantics are extremely well understood
and where a simple technical protocol for conversion to XML and
hence publication can be created. To support this part of the
data publication process we have created the XML-based Chemical
Markup Language (CML).5 The primary information
components (all of which are common and well understood)
are:

- **Molecular structure**. A compound is described by a
  compositional formula (e.g. CH3OH for methanol)
  and a graphical structural formula ("2D diagram"). These
  descriptions are required in bioscience publications for new
  compounds or where scientific arguments are based on details
  of chemical structure. There are a few widely used standalone
  tools (mainly commercial) for drawing structures and
  calculating certain properties. They output a variety of
  machine-processable formats (MDL Molfiles, ChemDraw CDX
  files, and increasingly CML). The main challenge is that the
  output is designed for the sighted human reader and that
  semantics may be implicit, discussed below. The Open Source
  community is creating tools (e.g. JChempaint,
  Jmol6) that will be valuable in authoring
  publications.
- **Chemical entities and names**. Many compounds have
  no explicit structures and are mentioned by names or
  identifiers. Where these relate to specific compounds (rather
  than generic such as "phenols") it is valuable to link them
  to a precise identification.
- **Spectra**. Many traditional formats (JCAMP and some
  manufacturers) are satisfactorily machine-processable, and we
  expect the XML-based AnIML7 to be widely adopted
  by manufacturers.
- **Crystal Structures**. Relatively few small-molecule
  crystal structures are reported in bioscience publications,
  but when they are we have a workflow-driven system that
  extracts the data automatically and reposits it
- **Molecular properties**. These are required as
  proof-of-synthesis and use a small dictionary of
  properties8. Their publication is highly
  ritualised and we expect that a publisher-wide template for
  the submission of this information would be straightforward
  to compile and welcomed by many authors.

### Identifying compounds

The identification of chemical entities is the most valuable
contribution that an author can make. In most cases s/he (as,
say, the purchaser or creator of the materials) is the best
judge of what was used . It is more considerably more difficult
to identify compounds after publication as we show below.

We list possible methods of publishing the identity of
compounds in machine-understandable form:

- **Connection table**. This is the most powerful method
  and we urge that every report of a chemical synthesis be
  accompanied by a connection table. It already exists in the
  authors' laboratory (in MOL, Chemdraw, SMILES, and
  increasingly as CML). It is rare that a pure molecule in the
  bioscience literature cannot be represented in this way. This
  is the single most important recommendation in this
  manuscript.
- **Chemical structure diagram**. This is a useful
  adjunct to a connection table (and some of the formats
  combine the two). Very occasionally (e.g. for catenanes,
  helicenes) a diagram is essential, but it should never be
  used instead of a connection table.
- **InChI**. For most compounds of bioscientific
  interest with known structure it is possible to generate a
  unique identifier using the new InChI9 from IUPAC.
  This has major advantages over non-semantic identifiers and
  Closed proprietary canonical identifiers such as
  SMILES10. In principle an InChI not only uniquely
  identifies the substance but also contains all the essential
  structural information. InChI (and other canonicalization
  schemes) start to break for metallic and ionic compounds but
  these limitations are unlikely to matter in bioscience.
- **Semantically free identifiers.**. These are provided
  by authorities (e.g. Chemical Abstracts, RTECS, PubChem,
  etc.). To be useful they should have an Open mechanism for
  their resolution (e.g. in PubChem), but this is often
  expressly forbidden. Thus Chemical Abstracts11
  forbids the public exposure of more than 0.1% of its content.
  Unless persistent Open machine-friendly resolution is
  available we deprecate the use of authority-controlled
  identifiers as unique IDs in primary publications. There are
  very few cases (e.g. zeolites) where identifiers are the best
  means of identification.
- **Trivial ("Common") names**. The structures of many
  compounds ("aspirin", "testosterone", "glycine"...) can only
  be found through lookup. In the past these names have been
  controlled in Closed collections but there are now an
  increasing number of Open lexicons of names:structures. The
  NCI led the way (220,000 names for ca 50,000 structures) and
  PubChem12 has continued to develop this. If
  commercial suppliers make their catalogs Open then most of
  the common chemical names in scientific discourse can be
  automatically linked to connection tables.
- **Systematic chemical names**. Until now this has been
  a common means of transmitting chemical identity, but it now
  serves little purpose, although It may be required legally,
  e.g. for patents. Most chemists would prefer a structural
  diagram to a systematic name, and many regard name generation
  as a tedious chore. In principle IUPAC2 chemical
  names obey a context-free grammar and there are complex rules
  for canonicalization. In practice most authors use a variety
  of shortcuts. This means that most compounds are reported
  with a variety of near synonyms (thus "2-hydroxy-toluene",
  "2-methyl-phenol" are semi-systematic variants for
  "1-hydroxy-2-methyl-benzene"). Free-text searching on
  chemical names has almost always low precision and often low
  recall. It is a common error to assume that deterministic
  grammars can parse any chemical name; in practice
  typographical errors, elisions and trivial fragments lower
  precision considerably. Some commercial tools are available
  but their algorithms are closed and little research has been
  done on their precision and recall. We suspect that they are
  composed of lexicons and heuristics but have no information
  on how they are maintained, especially in light of revisions
  of naming conventions.

### Issues with Chemical Names

Chemical names can be used with more or less specificity.
Thus "1,4-dichlorobenzene" is unambiguous in any context.
However there are several areas where more generic language is
used. This can arise because:

- The name refers to a class of compounds, whose members
  have similar structures and/or properties: "steroids", "amino
  acids", "monosaccharides", "polychlorinated biphenyls".
- The substance is a mixture of compounds: "60-80 petrol",
  "xylenes", "the phospho-inositols"
- The substance has not been fully identified: "the
  estradiol monobenzoate was ..." (there are two
  possibilities)
- The stereochemistry is ambiguous. The possibilities (in
  decreasing order of merit) include:
  - Stereochemistry is known and reported.
  - Stereochemistry is unknown and reported as such.
  - Stereochemistry is partially known and reported as
    such.
  - Stereochemistry is not reported but is unknown
  - Stereochemistry is not reported but is known
  - Stereochemistry is partially reported but is
    completely known
  - Stereochemistry is reported and is wrong

  "Glutamic acid" is an example of ambiguity through
  unspecified stereochemistry. Thus PubChem lists the three
  isomers (Table 1)

  A structure without any stereo information is more
  valuable than one with partial information of unknown
  quality. The InChI9 is an extremely powerful
  tool here. We have recently shown that "staurosporine"
  reported in publications and suppliers catalogs contains
  many instances of incorrect stereochemistry, and some
  partially correct and incorrect. Given that this is a
  single substance, its structure and absolute configuration
  has been known for a considerable time there is no reason
  for using any structure other than PubChem CID: 44259
- Ionization. Protons are labile in aqueous systems and (for
  example) aminoacids can have several ionization states. The
  importance of ionization details varies;
  - "Acetic acid (0.1M) was added..." [ionization state
    irrelevant]
  - Acetic acid forms a hydrogen-bonded dimer in the
    crystal. [single species, determinable by
    crystallography]
  - We computed the structure of glycine zwitterion acid
    (NH3+CH2CO2
    -) in the gas-phase [single species, distinct
    from NH2CH2CO2H]
  - "Glutamic acid is the most common excitatory
    neurotransmitter in the CNS" [irrelevant in macroscopic
    experiment, critical in modelling action at
    receptor]
- Tautomerism. Many neutral compounds, particularly with
  heteroatoms have mobile hydrogens in solution. Thus 2-hydroxy
  pyridine (Figure 1) exists as both forms with very rapid
  interchange. PubChem (as with many other systems) lists them
  as the same compound (CID8871) and gives the many synonyms
  including "2(1H)-Pyridinone" and "2-HYDROXYPYRIDINE".
  InChI9 has an extensive system for detecting
  tautomerism in compounds with heteroatoms, but does not yet
  address carbon compounds (e.g. CH2=CHOH as a tautomer of
  ethanal (acetaldehyde, CH3-CH=O).
- Imprecise or polysystemic use. This often occurs when a
  chemical entity is incorporated into a larger system
  - "This polysaccharide has a high mannose content"
    means "... contains many mannosyl fragments ..."
  - "HIV protease has a catalytic aspartic acid..." means
    "... an aspartyl residue ..."

The preceeding discussion shows how ambiguity and loss of
information can occur if structured procedures are not
followed. The following examples (Table 2) show some suggested
approaches to markup which can re-capture much of the
information loss described above.

The last example references a generic name, monosaccharide,
in the IUPAC guide2 to organic nomenclature with a
suggested use of identifiers.

## Case studies

In this second section, we take 3 articles from BMC
publications and show the success and problems of extracting
chemistry in machine-understandable form. These have been
randomly selected and do not necessarily reflect the average
quality of BMC publications. We note that in our other studies
of chemical text very few publications were error-free.

### Case Study 1: Identification of compounds in discourse and reagents in methods13.

The abstract is typical of the discourse:

> Background: Recent studies indicate that the G
> protein-coupled receptor (GPCR) signaling machinery can serve
> as a direct target of reactive oxygen species, including
> nitric oxide (NO) and S-nitrosothiols (RSNOs). To gain a
> broader view into the way that receptor-dependent G protein
> activation - an early step in signal transduction - might be
> affected by RSNOs, we have studied several receptors coupling
> to the Gi family of G proteins in their native cellular
> environment using the powerful functional approach of
> [35S]GTP?S autoradiography with brain cryostat sections in
> combination with classical G protein activation assays.  
> Results: We demonstrate that RSNOs, like
> S-nitrosoglutathione (GSNO) and S-nitrosocysteine (CysNO),
> can modulate GPCR signaling via reversible, thiol-sensitive
> mechanisms probably involving S-nitrosylation. RSNOs are
> capable of very targeted regulation, as they potentiate the
> signaling of some receptors (exemplified by the M2/M4
> muscarinic cholinergic receptors), inhibit others (P2Y12
> purinergic, LPA1lysophosphatidic acid, and cannabinoid CB1
> receptors), but may only marginally affect signaling of
> others, such as adenosine A1, µ-opioid, and opiate
> related receptors. Amplification of M2/M4 muscarinic
> responses is explained by an accelerated rate of guanine
> nucleotide exchange, as well as an increased number of
> high-affinity [35S]GTP?S binding sites available for the
> agonist-activated receptor. GSNO amplified human M4 receptor
> signaling also under heterologous expression in CHO cells,
> but the effect diminished with increasing constitutive
> receptor activity. RSNOs markedly inhibited P2Y12 receptor
> signaling in native tissues (rat brain and human platelets),
> but failed to affect human P2Y12 receptor signaling under
> heterologous expression in CHO cells, indicating that the
> native cellular signaling partners, rather than the P2Y12
> receptor protein, act as a molecular target for this
> action.  
> Conclusion: These in vitro studies show for the first time
> in a broader general context that RSNOs are capable of
> modulating GPCR signaling in a reversible and highly
> receptor-specific manner. Given that the enzymatic machinery
> responsible for endogenous NO production is located in close
> proximity with the GPCR signaling complex, especially with
> that for several receptors whose signaling is shown here to
> be modulated by exogenous RSNOs, our data suggest that GPCR
> signaling in vivo is likely to be subject to substantial, and
> highly receptor-specific modulation by NO-derived RSNOs.

The above contains reference to a considerable numbers of
individual compounds. The authors helpfully publish a table of
abbreviations to assist in the compound identification process
(Figure 2).

Using this as our data, we have attempted to identify (Table
3) the "small-molecules" mentioned in the discourse. Using
PubChem and occasional suppliers catalogs, the elapsed real
time was about 1 hour. It can be seen that of 19 molecules, 15
were identified without problems or error, 2 were not (CysNOGly
and Glu-CysNO) and 2 required additional expertise by the
reader. We estimate that it would take an author the same
amount of time to add PubChem IDs for novel compounds and much
less time if they were in common use in their laboratory.

It is only a little additional effort to convert each
molecule to a more formal description expressed in e.g.
CML5 and which can carry not only an atom connection
table and the corresponding InChI identifer, but also molecule
"meta-data" describing the provenance of the information:

```
<cml:molecule xmlns:cml="http://www.xml-cml.org/schema/cml2/core" title="carbacholine">
<cml:metadataList title="generated automatically from Openbabel">
<cml:metadata name="dc:creator" content="OpenBabel version 1-100.1"/>
<cml:metadata name="dc:description" content="Conversion of legacy filetype to CML"/>
<cml:metadata name="dc:identifier" content="InChI"/>
<cml:metadata name="dc:content"/>
<cml:metadata name="dc:rights" content="open"/>
<cml:metadata name="dc:type" content="chemistry"/>
<cml:metadata name="dc:contributor" content="rzepa"/>
<cml:metadata name="dc:creator" content="Openbabel V1-100.1"/>
<cml:metadata name="dc:date" content="Tue May 17 12:02:50 BST 2005"/>
<cml:metadata name="cmlm:structure" content="yes"/>
</cml:metadataList>
<cml:identifier convention="iupac:inchi">InChI=1/C6H14N2O2.ClH/c1-8(2,3)4-5-10-6(7)9;/h4-5H2,1-3H3,(H-,7,9);1H</cml:identifier>
<cml:atomArray atomID="a1 a2 a3 a4 a5 a6 a7 a8 a9 a10 a11 a12 a13" 
     elementType="N C C O C O N C C C H H Cl" 
     formalCharge="1 0 0 0 0 0 0 0 0 0 0 0 -1" 
     x2="-1.892900 -1.178500 -0.464000 0.250500 0.964900 0.964900 1.761800 -2.305400 -2.476300 -1.480400 2.174300 2.476300 -1.921800" 
     y2="0.415300 0.827800 0.415300 0.827800 0.415300 -0.409700 0.628800 1.129800 -0.168000 -0.299200 1.343300 0.216300 -1.343300"/>
<cml:bondArray atomRef1="a1 a1 a1 a1 a2 a3 a4 a5 a5 a7 a7" 
               atomRef2="a2 a8 a9 a10 a3 a4 a5 a6 a7 a11 a12" 
               order="1 1 1 1 1 1 1 2 1 1 1"/>
</cml:molecule>
```

Such molecular datuments can be embedded in any XML-based
document in a manner which can if needed survive e.g. journal
production processes, and where the molecular information can
be extracted and re-used at any stage.

### Case Study 2: Identity and properties of synthesised compounds14

Our critique of the chemistry requires context, given by the
abstract:

> Abstract Background: Kynureninase is a key enzyme on the
> kynurenine pathway of tryptophan metabolism. One of the end
> products of the pathway is the neurotoxin quinolinic acid
> which appears to be responsible for neuronal cell death in a
> number of important neurological diseases. This makes
> kynureninase a possible therapeutic target for diseases such
> as Huntington's, Alzheimer's and AIDS related dementia, and
> the development of potent inhibitors an important research
> aim.  
> Results: Two new kynurenine analogues,
> 3-hydroxydesaminokynurenine and 3- methoxydesaminokynurenine,
> were synthesised as inhibitors of kynureninase and tested on
> the tryptophan-induced bacterial enzyme from Pseudomonas
> fluorescens, the recombinant human enzyme and the rat hepatic
> enzyme. They were found to be mixed inhibitors of all three
> enzymes displaying both competitive and non competitive
> inhibition. The 3-hydroxy derivative gave low Ki values of 5,
> 40 and 100 nM respectively. [...]  
> Conclusion: For kynureninase from all three species the
> 2-amino group was found to be crucial for activity whilst the
> 3-hydroxyl group played a fundamental role in binding at the
> active site presumably via hydrogen bonding. The potency of
> the various inhibitors was found to be species specific. The
> 3-hydroxylated inhibitor had a greater affinity for the human
> enzyme, consistent with its specificity for
> 3-hydroxykynurenine as substrate, whilst the methoxylated
> version yielded no significant difference between bacterial
> and human kynureninase. [...]

We note that "quinolinic acid" has 4 mentions in the text,
but its formula is not given. We took 2.7 minutes to identify
CID1066 in PubChem, with the additional useful information
(from Medline/MeSH):

> A metabolite of tryptophan with a possible role in
> neurodegenerative disorders. Elevated CSF levels of
> quinolinic acid are correlated with the severity of
> neuropsychological deficits in patients who have AIDS

The name "3-hydroxydesaminokynurenine" [the synthesized
compound (4)] presents a more serious problem. Although the
structure is given in a diagram, the stereogenic centre is not
marked. It would be a reasonable assumption that "kynurenine"
refers to a natural product which is only found in one
enantiomeric form and "desamino" was also chiral. Careful
reading (requiring chemical expertise) showed that the authors
had probably synthesised a racemic mixture, since they started
with achiral compounds and did not report chiral reagents or a
resolution step. The compound should have been reported as
(R/S)-3-hydroxydesaminokynurenine or (much better) as the
IUPAC-like name "IUPAC Name: (R/S)
2-amino-4-(3-hydroxy-phenyl)-4-oxo-butanoic acid". Indeed many
referees and editors would have insisted on this specification.
In the event, as we show below, this is not the reported
compound!

The tools we are proposing would immediately have queried
both these concerns *at time of authoring* and, had they
been available to the technical editor would have produced a
more useful and more easily readable paper.

The publication of the synthesis or re-synthesis of
compounds must be accompanied by analytical and property data
to prove purity and identitity. The ritualistic presentation
shown below (Figure 3) as copied from the manuscript is
entirely typical of most chemical publications. Note that it is
visually challenging to read and this is entirely due to the
publisher's requirements of using a system designed to save
paper rather than communicate useful information.

For each compound this compressed information is (manually)
created from some or all of:

1. An elemental analysis (probably in machine-understandable
   form)
2. A calculated composition for the compound (machine
   understandable)
3. An infrared spectrum (machine understandable)
4. A 1H spectrum (machine understandable)
5. A 13C spectrum (machine understandable)
6. A low resolution mass spectrum (machine
   understandable)
7. A high resolution mass spectrum (machine
   understandable)

For the publication, the authors have to measure peak
heights from the spectrum (possibly with a ruler), and
transcribe them to a Word or PDF format, probably by typing the
values or cut-n-pasting them. We have developed an Open Source
robot (OSCAR)8 which can understand this data if it
is syntactically correct, and the result is shown in Figure
4:

The coloured parts are those that adhere to the publication
guidelines. We found 7 changes that had to be made to the
punctuation (missing punctuation, syntactic variation is common
in many chemical papers). OSCAR can then understand and check
the data. For compound [4] it announces

```
There are fewer H atoms by NMR integration (7) than there are by elemental analysis (12)
```

This is acceptable because there are exchangeable groups.
However it also announces:

```
There are more C-NMR environments (11) than there are C atoms from elemental analysis (10).
```

as it found the string "114.47 120.78". We also do not
understand this and it may be an error (or it could be a
solvent peak or other impurity). OSCAR also had problems
interpreting the chemical formula:
"C11H14NO4" which in fact
turns out to be a charged species. In fact the compounds are
poorly identified. They appear to be not the aminoacids
"3-Hydroxydesaminokynurenine (4)" and
"3-Methoxydesaminokynurenine (5)" but their hydrochloride
salts. This is not a trivial error; the melting points and
infrared spectra of the parents and their salts will be
significantly different and would cause errors if transcribed
unthinkingly from the paper.

Even with OSCAR it took one of us ca 45 minutes to make sure
that the above analysis was correct. From several anecdotal
conversations with typical authors we estimate that it took
about 2 hours to prepare this part of the submission; a
thorough reviewer might take 0.5 hour to decipher it. All of
this is unnecessary if the original connection tables, spectra
and analytical data were made available in uncorrupted form. As
it is, much of the original data is lost; using the reported
peaks OSCAR does its best to recreate what the spectrum might
have looked like (Figure 5). Precise peak shapes and traces of
impurities are lost in this representation.

### Case Study 3. Identity of compounds and preservation of calculations15

Here too a number of small-molecules are reported without
formulae;

> Background [...] Phenols and anilines are generally
> recognized as substrates of the heme peroxidases (donor: H2O2
> oxidoreductases EC 1.11.17). The peroxidases catalyze
> oxidation of the substrates by hydrogen peroxide or alkyl
> peroxides, usually but not always, via free-radical
> intermediates [1,2]. Nonphenolic compounds, such as
> indole-3-acetic acid, phenylenediamines, ferrocenes,
> phenothiazines, phenoxazines, have also been investigated as
> peroxidase substrates [2][3-5]. Steady-state kinetics of
> peroxidase action has been described as a ping-pong scheme
> with compound I and compound II formation [1].

This paper also has issues with the identity of
compounds.

This is again a visually unacceptable format dictated by the
prevailing business model of chemical publishing. Note
"Napthyl" is misspelt, presumably because it has been
(mis)typed by the authors, which would give unnecessary
problems to chemical text-mining robots. Worse, the identity of
AHA5 is genuinely unclear, in that the connection could be to
either of the phenyl groups in the fragment: "Ph-C(O)N(OH)-Ph".
BHA (also described elsewhere by "benzhydroxamic acid") has no
structural or compositional formula. Worse, BHA in the PDB
ligand collection refers to 2-hydroxy-4-amino-benzoic acid (a
completely different compound); "benzhydroxamic acid" has code
BHO.

Another section of this article describes various
computational modelling techniques applied to these molecules;
here we can assume that the authors had precise coordinates for
all the computed species available at the end of the research,
although none of this data is actually made available via the
final published article. Some of this data is used to drive a
docking program, which itself implies a protocol used to
specify various run-time parameters. Some of these are declared
in the article, many probably default to values set internally
within the program. There are also ambiguities in the declared
computational protocol:

> The optimized geometry of molecules was used for energies and
> charges calculations with a 6-31G basis set using RHF and
> B3PW91 (Density Functional Theory).

Here, the RHF and the B3PW91 protocols are mutually
exclusive; either one or the other could have been used, but
not in combination. Mapping either protocol to e.g. the
appropriate input for the program package used can also be a
challenge for anyone not totally familiar with the program;
program manuals are still designed largely for human rather
than machine use. Such ambiguities, and lack of data, make
repetition of the modelling more difficult for others.

## A Proposed infrastructure

It should now be clear that the current system of
communicating chemistry (which is common to all publishers and
all disciplines) is inefficient, costly, lossy, and of
questionable quality. We present a new XML-based approach which
we show:

- takes less time
- conveys more information
- is easier to read
- allows published data to be aggregated and re-used

We note that when starting to draft a publication the author
*already has*

- **free text (A)** (probably in handwritten form)
- **properties (B)** (probably handwritten form)
- **spectra (C)** (probably in digital form)
- **molecules (D)** (probably in MOL or ChemDraw
  files)

Electronic lab notebook technology is not well advanced in
chemistry; our architecture would provide a good method for
preserving conventional data. It looks as shown in Figure 7
(blue = XML):

The author would then use a tool which can manage structured
XML documents and provide normal textual support (spellchecks,
etc.). There are 4 additional tools required to support
chemical information:

- **A**. Chemical lexical tool(**AA**) which can (a)
  parse free text**(A**) for possible compound names (b)
  look them up or (c) parse them to create connection table
  and (d) insert a reference (**AX**) to the lexicon in
  the text, e.g.:

  ```
  ... When foobarone is broken down, the presence of indole can be detected ...
  ```

  might be marked up as

  ```
  ... When <cml:molecule name="foobarone" dictRef="natprod:foobarone"/> is broken down, the presence of <cml:molecule>
  <identifier convention="iupac:inchi" title="indole">1/C8H7N/c1-2-4-8-7(3-1)5-6-9-8/h1-6H,9H</identifier>
  </cml:molecule>
  indole can be detected ...
  ```
- **B**. A controlled vocabulary (**BB**) of property
  types is used in a template to capture properties
  (**B**) and create a CML table (**BX**), e.g.

  ```
  yield(93%), M.Pt. 273-275oC
  ```

  becomes

  ```
  <cml:list>
    <cml:property dictRef="cml:yield">
      <cml:scalar units="cml:percent">93%lt;/cml:scalar>
    </cml:property>
    <cml:property dictRef="cml:mpt">
      <cml:scalar units="cml:celsius" minValue="273" maxvalue="275"/>
    </cml:property>
  </cml:list>
  ```
- **C**. Spectra in legacy format (**C**) are
  automatically converted to CMLSpect or AniML
  (**CX**).
- **D**. Molecules created in a conventional editor are
  either emitted in CML (**DX**) or automatically converted
  from legacy (**D**) .

The result is a single structured XML
"datument"16 containing fine-grained markup of facts
(molecules, measurements, properties, chemical names). This
datument can be used to create derivatives such as the
"full-text" or the "supplemental data". The complete datument
(if Open) or the "data" if not is then reposited (**XX**)
where it can be harvested. New compounds with their names are
fed back into the lexicon and all compound/property data is
available for datamining and computational re-use (e.g. for
further *in silico* prediction. A human or robot reader
has access to the same lexicons and dictionaries as the author
so that the semantics and ontology of authoring are the same as
those of reading (and of preservation).

### Metadata and Rights

The social aspects of metadata and rights were addressed in
(1). To meet these we place special emphasis on the XML and its
metadata. Fine-grained XML (e.g.
<scalar>...</scalar> or
<molecule>...</molecule> represents facts which can
be identified as Open and not the property of the publisher.
Hyperlinks and structure for semantics (e.g. identification of
compounds in PubChem) are also Open. Tools such as XSLT can
then extract the factual, non-copyrightable information with
little technical problem. Rights should be explicitly marked
up. If the publisher supports Open Access and also Open Data
then it is valuable to label the appropriate components with
Open licenses, such as the RDF metadata provided by Creative
Commons. It is also possible to preserve authors' moral rights
and provenance of data re-used within the paper (e.g. spectra
of molecules or coordinates of protein structures).

## Realising the vision

The transition to this architecture will have a cost, so
short term-benefits are particularly attractive. Moreover most
of the parties are not used to a communal approach (pressures
are normally per-institution and per-publisher).

### Costs

- Time lost in understanding and changing to a new
  system.
- New tools might cost money, or have to come from
  non-centralised budgets
- Only supported by a subset of publishers
- Communal deposition of data goes against the secretive
  culture
- Publishers have to invest in new system and react to
  community expectations

### Benefits

- Open Access and Open Data18
- Greater quality in publications
- Data in theses and papers can be interchanged
- Greater readability, usability and innovation in
  publications
- Repository provides complete data record for laboratory,
  institution and world
- Modern informatics tools allow new types of search and
  aggregation
- Considerable time-savings during publication
- More efficient publishing reduces author frustration and
  time to publication
- and most importantly the arrival of the Scientific
  Semantic Web, allowing robots to read and take action on
  publications.

The benefits should also be clear for most individuals and
organisations:

- **funders** can ensure a much higher of dissemination
  of funded data will be available
- **institutions** mandate a greater proportion of
  funded science published; better visibility and
  preservation
- **researchers** spend less time on unproductive
  operations
- **reviewers** have easier access to background
  ontology of data in documents
- **editors** get greater automation
- **publishers** are relieved of need to archive
  supplemental data
- **readers** have information prosthetics for easier
  and faster reading
- **librarians** develop one of the best early
  repository applications in the digital age

### Potential

Because the chemical information is structured we now have a
*biocheminformatics* cycle (this term - with spelling as
here - is in modest use. We suggest its adoption to describe
the management of chemical information in biosciences and not
just in biochemistry) where, for the first time, large scale
robotic data analysis can take place (Figure 8).

The data in the research (laboratory, *in silico*, or
both) are published in a lossless manner. Molecules and their
properties have unique identifiers as described above and can
be integrated into mainstream bioinformatics in the same manner
as collections such as PubChem, MSDChem (at EBI), KEGG, etc.
They will bring the added value of consistently captured
property data and spectra. We also expect that many *in
silico* properties will then be systematically added.

### Compliance and adoption

The introduction of structured authoring tools will help
this process considerably. Templates can be created for the
chemical components described above and where the information
exists in XML (connection tables, spectra, properties) it
should be as easy as for committed authors as using a
semantically void tool (e.g. Word). Where information needs to
be converted from legacy formats we have created Open Web
Services which publishers (and authors) may clone and
customise. The main technical challenge will be the management
of chemical names in free text.

## Conclusions and the future.

The analysis presented here introduces the basic concepts of
chemistry in bioinformatics. Many areas remain to be addressed;
we briefly describe two below which have immediate
application.

### Reactions

Chemical reactions are very patchily abstracted from the
literature and the products are almost always closed. The
motivation for the primary publication of reactions in
bioscience includes:

1. record of synthesis of compound and proof thereof
2. record of an experimental protocol (e.g.
   *biotinylation*)
3. record of a biochemical reaction, including xenobiotic
   processes
4. description of systems biochemistry (coupled reaction
   pathways)
5. understanding of an enzyme mechanism

CMLReact (an extension of CML) has been created19
to support these catagories of reaction. Items 1-2 require
identical support as in mainstream chemistry (e.g. in journals
supporting organic synthesis). Item 3 can be supported by
CMLReact though there is little current experience. Item 4 is
supported by SBML20 and efforts such as
BioPAX21 (in which CML is a tool). Item 5 is
particularly exciting and exemplified by our MACiE
database22 where 150+ enzymes with 3D structures and
proposed mechanisms have been collected. Currently the
abstraction is manual and expensive, but if the ideas in the
current paper are implemented we shall present an extension
whereby mechanisms can be relatively cheaply captured at
source. This would be a major new resource in
bioinformatics.

### Evaluation metrics

The primary motivation for a publication, of course, is
citability and the technology we describe raises the fear among
chemists that the data in it might actually be read, analysed
and re-used. However it also raises the vision of changing the
"citation economy" (which values market perception) to a "reuse
economy" where a the data in an article (or as we prefer, a
"datument") are valued by how often they are re-used.

## Notes and references

1. Murray-Rust P, Mitchell JBO, Rzepa HS, *BMC
   Bioinformatics*, 2005, **6**:XXX.
2. The International Union of Pure and Applied Chemistry;
   1919-present. http://www.iupac.org/
3. Murray-Rust P, Rzepa HS, Tyrell SM, Zhang Y: *Org.
   Biomol. Chem.*, 2004, **2**:3192-3203.
4. For information on this infrastructure, see http://www.w3c.org/
5. Murray-Rust P and Rzepa HS, *J. Chem. Inf. Comp.
   Sci.*, 2003, **43**:757-772. See also http://cml.sourceforge.net/
6. Much of this OpenSource software is hosted at the
   Sourceforge site. See Jchempaint: http://jchempaint.sourceforge.net/
   ; Jmol: http://jmol.sourceforge.net/
7. Kramer, GW *Abstracts of Papers, 226th ACS National
   Meeting,* New York, NY, United States, September 7-11,
   2003, CINF-080. See also http://animl.sourceforge.net/
8. Townsend JA, Adams SE, Waudby CA, de Souza VK, Goodman
   JM, Murray-Rust, P, *Org. Biomol. Chem.* 2004,
   **2**:3294-3300.
9. See http://www.iupac.org/inchi/
   and also http://inchi.sourceforge.net/
10. See http://www.daylight.com/smiles/f\_smiles.html
11. See for example http://www.scienceip.org/data\_use\_restrictions.html
    A User or Organization may include, without a license and
    without paying a fee, up to 10,000 CAS Registry Numbers or
    CASRNs in a catalog, website, or other product for which
    there is no charge. The following attribution should be
    referenced or appear with the use of each CASRN: CAS Registry
    Number® is a Registered Trademark of the American
    Chemical Society. CAS recommends the verification of the
    CASRNs through CAS Client ServicesSM
12. See http://pubchem.ncbi.nlm.nih.gov/
13. Kokkola T, Savinainen JR, Mšnkkšnen KS,
    Retamal, MD, Laitinen JT, "S-Nitrosothiols modulate G
    protein-coupled receptor signaling in a reversible and highly
    receptor-specific manner", *BMC Cell Biology*, 2005,
    **6**:21 doi:10.1186/1471-2121-6-21. http://www.biomedcentral.com/1471-2121/6/21
14. Walsh HA, O'Shea KA, and Bottin NP, "Comparative
    inhibition by substrate analogues 3-methoxy- and
    3-hydroxydesaminokynurenine and an improved 3 step
    purification of recombinant human kynureninase." *BMC
    Biochemistry*, 2003, **4**:13.
    http://www.biomedcentral.com/1471-2091/4/13
15. Kulys J, and Ziemys A, "A role of proton transfer in
    peroxidase-catalyzed process elucidated by substrates docking
    calculations", *BMC Structural Biology*, 2001,
    **1**:3. http://www.biomedcentral.com/1472-6807/1/3
16. Murray-Rust P and Rzepa HS, *J. Digital Inf.*, 2004,
    **5**:248.
17. See http://www.soros.org/openaccess/
18. "Open Data" is not a widely used concept. We are
    preparing a discussion document for public debate of this
    concept.
19. Holliday, GL, Murray-Rust P and Rzepa HS, *J. Chem.
    Inf. Mod.*, 2005, submitted for publication.
20. See http://sbml.org/
21. See http://www.biopax.org/
22. Holliday, GL, Bartlett GJ.; Murray-Rust P, Thornton, JM,
    Mitchell, JBO. *Abstracts of Papers, 226th ACS National
    Meeting, New York, NY, United States,*, September 7-11,
    2003, CINF-099. See also 
    http://www-mitchell.ch.cam.ac.uk/macie/MACiEDictionary.html

## Figures

- **Figure 1**. Tautomers of Hydroxypyridine.
- **Figure 2**. Abbreviations used in reference 13.
- **Figure 3**. A linear text-based description of
  experimental detail and data taken from Ref. 14.
- **Figure 4**. OSCAR output from the text-based
  description in Ref 15.
- **Figure 5**. OSCAR generated spectrum of analytical
  information reported in Ref 15.
- **Figure 6**. Structure diagram reported in Ref 16.
- **Figure 7**. Data-flow illustrating the use of XML.
- **Figure 8**. A Biocheminformatics Cycle.

## Tables

- | Table 1. Isomers of Glutamic acid | |
  | --- | --- |
  | CID | name(s) |
  | 611 | glutamic acid |
  | 33032 | L-glutamic acid |
  | 23327 | D-glutamic acid |

  - | Table 2. Examples of approaches to chemical Identification. | | |
    | --- | --- | --- |
    | Prose description | More precise suggested naming using the CML5 approach | Type of information |
    | *Acetaldehyde* has a general narcotic action | <p><cml:molecule> <cml:identifier convention="iupac:inchi">1/C2H4O/c1-2-3/h2H,1H3</cml:identifier> <cml:identifier convention="pubchem:CID">177</cml:identifier> </cml:molecule> has a ...</p> | precise, redundant |
    | *Benzo(a)pyrene* is a potent mutagen and carcinogen | <p><cml:molecule><cml:identifier convention="pubchem:CID">2336</cml:identifier></cml:molecule> is a ...</p> | precise |
    | *glycine* (1mmol) was added ... | <p><cml:molecule title="glycine"><cml:identifier convention="iupac:inchi">1/C2H5NO2/c3-1-2(4)5/h1,3H2,(H,4,5)</cml:identifier></cml:molecule> is a ...</p> | hydrogens mobile |
    | calculations on *glycine zwitterion*... | <p><cml:molecule title="g><cml:identifier convention="pubchem:CID">InChI=1/C2H5NO2/c3-1-2(4)5/h1H3,3H2</cml:identifier></cml:molecule> is a ...</p> | hydrogens precise |
    | ... a monosaccharide transporter... | <p>a <cml:molecule title="monosaccharide"><cml:identifier convention="iupac:carbohydrate">2-Carb-2</cml:identifier></cml:molecule> transporter ...</p> |

    - | Table 3. Identification of Small-molecules noted in Ref. 14 | | | |
      | --- | --- | --- | --- |
      | abbrev | author name | PubChem ID | Notes |
      | 2MeSADP | 2-methylthio-ADP | [121990] | Not found directly in PubChem. Located in supplier's catalog. Synonym from that found in PubChem |
      | 5-HT | 5-hydroxytryptamine | 5202 |  |
      | CCh | carbacholine | 521353 |  |
      | CP-55940 | (-)-3-[2-hydroxy-4-(1,1-dimethylheptyl)-phenyl]-4-[3-hydroxypropyl]cyclohexan-1-ol | 104895 | IUPAC: 5-(1,1-dimethylheptyl)-2-[5-hydroxy-2-(3-hydroxypropyl)cyclohexyl]-phenol |
      | CysNO | S-nitrosocysteine | 39933 |  |
      | CysNOGly | S-nitroso-cysteinyl-glycine |  | Text search on PubChem found wrong compound. Not found in major supplier |
      | DAMGO | [D-Ala2, N-Me-Phe4, Gly5-ol]-enkephalin | 104742 |  |
      | DPCPX | 8-cyclopentyl-1,3-dipropylxanthine | 1320 |  |
      | DTT | dithiotreitol | 19001 |  |
      | Glu-CysNO | L-?-glutamyl-S-nitrosocysteine |  | Identity unresolved |
      | GSH | glutathione | 745 |  |
      | GSNO | S-nitrosoglutathione | 104858 |  |
      | LPA | lysophosphatidic acid | 3987 |  |
      | NA | noradrenaline | 951 | PubChem CID covers both racemic and d-enantiomer |
      | NO | nitric oxide | 84878 | PubChem also lists 945 (with incorrect formula HNO) as nitric oxide |
      | NOBF4 | nitrosodium tetrafluoroborate | 151929 | Paper has a typographical error for "nitrosonium". Structure in PubChem is wrong (formula should be NO+BF4-, not H2NO+.BF4-) |
      | SNAP | S-nitroso-N-acetyl-D,L-penicillamine | 5231 | PubChem does not list stereochemistry |
      | RSNO | S-nitrosothiol |  | Appears to be a generic compound (R-S-N=O) |
      | SNP | sodium nitroprusside | 26256 |  |
